# Predictive Factors of Survival and 6-Month Favorable Outcome of Very Severe Head Trauma Patients; a Historical Cohort Study

**Published:** 2017-01-10

**Authors:** Karin Vathanalaoha, Thakul Oearsakul, Thara Tunthanathip

**Affiliations:** Neurosurgical unit, Department of Surgery, Faculty of Medicine, Songklanagarind Hospital Prince of Songkla University, Hat Yai, Songkhla, Thailand.

**Keywords:** Glasgow coma scale, head injuries, closed, prognosis, treatment outcome, outcome assessment

## Abstract

**Introduction:**

Very severe head trauma cases, defined as Glasgow Coma Scale (GCS) scores of less than 6, have a higher mortality rate and poorer outcome. The purpose of this study was to recognize factors associated with survival and 6-month favorable outcome of very severe head trauma patients presenting to emergency department.

**Methods:**

In this historical cohort study, the authors retrospectively reviewed medical records of head trauma patients who were admitted to the emergency department with post-resuscitation GCS scores of less than 6. Both univariate and multivariate analyses were used to test the association between various parameters with survival and 6-month outcome.

**Results:**

103 cases with the mean age of 39 ± 16.5 years were studied (80% male). The overall survival rate was 41.7% and the rate of 6-month favorable outcome was 28.2%. In multivariate analysis, brisk pupil light reaction on admission and patent basal cistern on brain computed tomography (CT) scan were significant factors associated with both survival (OR 5.20, 95% CI 1.57-17.246, p = 0.007 and OR 3.65, 95% CI 1.22-10.91, p=0.02 respectively) and favorable outcome (OR 4.07, 95% CI 1.35-12.24, p=0.01 and OR 3.54, 95% CI 1.22-10.26, p 0.02), respectively.

**Conclusion:**

Based on the results of present study, the survival rate of patients with very severe head trauma (GCS < 6) was 41.7%. The strong predictors of survival and 6-month favorable outcome of these patients were brisk pupillary reactivity and patent cistern on brain CT scan. It seems that very severe head trauma patients still have a reasonable chance to survive and aggressive management should be continued.

## Introduction

Accidents are among the top three causes of death in Thailand. The incidence rate of traffic accident mortality in Thailand was 21.61-23.16 per 100,000 people and the trend of mortality gradually increased between 2010 and 2013. Pheunpathom et al. reported that 15.8-29% of trauma patients in the emergency room had head injuries and the most common mechanism of head injury was road traffic accidents (1). 

Generally, the severity of head injury was categorized into 3 categories using Glasgow Coma Scale (GCS) scores, mild head injury (GCS scores 15-13), moderate head injury (GCS scores 12-9) and severe head injury (GCS scores 8-3). Gennarelli et al. reported that patients with GCS scores of 3 to 5 had a higher mortality rate than patients with GCS scores of 6 to 8 (2). Very severe head injury was defined as cases of head trauma with GCS scores of less than 6 (3) because the highest mortality rates have been reported 42-87.5% in this group (2-4). These patients were subject to limited use of resources and even treatment in several facilities (3, 5-7). 

A small number of studies have involved patients with GCS scores <6 and have reported that, <60 years of age, adequate spontaneous respiration, brisk pupillary reactivity, increase in GCS score by at least 2 within 24 hours of admission and the appearance of basal cistern on first brain computed tomography (CT) scan were significant predictors of outcome in very severe head injury (3-8). 

The purpose of this study was to recognize factors associated with survival and 6-month favorable outcome of very severe head trauma patients presenting to emergency department.

## Methods

***Study design***

In this historical cohort study, the authors retrospectively reviewed computer-based medical records of all head trauma patients who were admitted to Songklanagarind Hospital between January 1, 2008 and March 31, 2011. Songklanakarind Hospital is the tertiary care center serving both direct transfers from the accident scene and transfer from 14 provinces in the Southern region of Thailand (1). The authors adhered to all principles of Helsinki declaration and confidentiality of patient records during the study period. The Human Research Ethics Committee of the Faculty of Medicine, Songklanagarind Hospital, Prince of Songkla University approved the study. 

***Participants***

The patients who had post-resuscitation GCS scores of less than 6, which persisted for at least 6 hours were selected to participate in the present study. However, patients with penetrating mechanism and pediatric cases were excluded. Documented data including the demographic data, mechanism of injury, physical examination, neurological examination, neurological imaging findings, types of intracranial hematoma, treatments, and types of surgery were gathered from computer-based medical records by a neurosurgeon. Additionally, the 6-month outcome of injury was documented by telephone call with patient/caregiver. Also, brain CT scans of all patients were assessed regarding intracranial lesions and the appearance of the basal cisterns according to the Marshall classification (9) by a neuro-radiologist. 

***Definitions***

The pupillary reaction to light on admission was classified into 2 groups; the poor pupils group included patients who had sluggish/no reactivity to light on both sides and with any size of pupils, while the brisk pupil/pupils group consisted of those who had at least one side of pupil to briskly response to light.

Glasgow Outcome Scale (GOS) was used for outcome measurements. GOS consists of good recovery (GR), moderated disability (MR), severe disability (SD), vegetative stage (VS), and death (D) (10). Finally, based on GOS, patients were dichotomized into 2 groups. The favorable outcome group included GR and MR, while the unfavorable outcome group consisted of all others. 

The Marshall classification categorized brain CT scans into 6 groups using some intracranial features. This system is composed of diffuse injury I (no visible intracranial pathology), diffuse injury II (cistern are present with midline shift < 5 mm, and no high/mixed density lesion > 25 cm^3^), diffuse injury III (basal cisterns compressed or absent with midline shift 0-5 mm, and no high/mixed density lesion > 25 cm^3^), diffuse injury IV (midline shift > 5 mm, no high/mixed density lesion > 25 cm^3^ ), evacuated mass (V) (any lesion surgically evaluated), and non-evacuated mass lesion (VI) (no high/mixed density lesion > 25 cm^3 ^and not surgically evacuated) (9).

Motor response is one of the three tests in Glasgow Coma Scale. The motor response is comprised of 6 grades: obeys commands (M6), localizes to pain (M5), withdrawal from pain (M4), decorticate posturing (M3), decelerate posturing (M2), and no motor response (M1) (11). 

***Statistical analysis***

All analyses were performed by the R and Epical program 2.9.1 version (12).Mean and standard deviation were calculated for descriptive purposes. The Chi-square test and Fisher’s extract test were performed for comparison of dichotomous parameters between groups. Univariate logistic regression analysis was used to compare the odds of several parameters to predict the outcome and survival. Finally, multivariate logistic regression analysis was used to adjust the odds to predict outcome and survival of the patients. A p<0.05 was considered statistically significant. 

## Results

***Demographic data***

103 cases with the mean age of 39 ± 16.5 years (range: 16.5-82) were studied (80% male). Table 1 shows the demographic data and baseline characteristics of the patients. Two-third of patients had GCS 3. The median time of pre-hospital transfer was 30 minutes (IQR 20, 90) and pre-hospital hypoxia and hypotension were observed in 17.5% and 44.7%, respectively. Seventy-nine individuals (76.7%) were in poor pupil groups. The median time of injury to brain CT scan was 77.5 minutes (IQR 41.2). The most common brain CT scan findings were subdural hematomas (75.7%), calvarial skull fracture (58.3%), basal skull fracture (59.2%), and basal cistern obliteration (56.3%). Two-third of patients (65.4%) were admitted to the intensive care unit (ICU) and the remainder were admitted to the trauma (28.2%) or neurosurgical units with intensive care (6.4%). During acute hospitalization, the median length of hospital stay was 9 days (IQR 2, 29 days). Forty-seven individuals (45.6%) required emergency surgery and decompressive craniotomy with clot removal was the most common surgical procedure that the patients underwent. 

***Survival rate and outcome***

The overall survival rate in the present study was 41.7%. Twenty-seven patients (26.2%) observed desirable recovery after acute hospitalization and 2 patients recovered to a favorable group by 6 months after injury. On the other side, sixteen patients (15.6%) were dependent and no patient died during the 6-month follow-up. 

***Factors associated with survival rate***

Table 2 shows the univariate analysis to identify factors associated with survival rate. The authors found that brisk pupillary reactivity, hypotension on admission, hydrocephalus, patent basal cistern, Marshall Classification, and performing operation were factors that associated with survival in univariate analysis. Table 3 shows the results of multivariate analysis in this regard. Brisk pupillary reactivity and patent basal cistern on brain CT scan were factors that significantly associated with survival.

***Factors associated with 6-month favorable outcome***

Table 4 shows the univariate analysis to identify factors associated with 6-month favorable outcome. Brisk response to light of pupil or pupils, epidural hematoma thickness less than 1.5 cm, patent basal cistern and Marshall Classification were factors associated with 6-month favorable outcome in univariate analysis. Table 5 reveals the results of multivariate logistic analysis in this regard. Accordingly, brisk pupillary reactivity and patent basal cistern on brain CT scan had a statistically significant association with 6-month favorable outcome. 

***Survival classification tree***

**Figure1:**
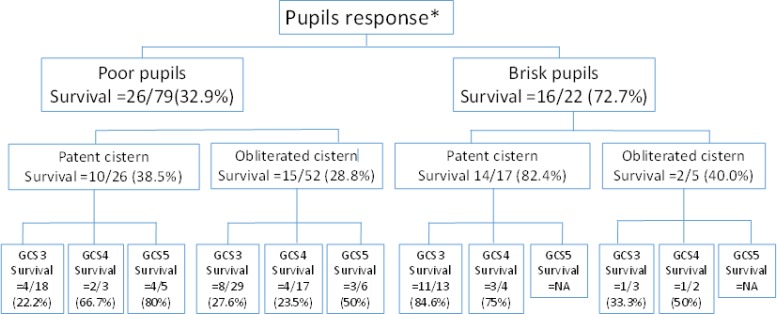
Classification tree for predictive variables associated with survival.

Figure 1 describes the survival chances of very severe head trauma patients in a classification tree. A higher survival rate was observed in the group with GCS scores of 5 compared to patients with GCS scores of 3-4 when they had similar poor pupil response and obliterated cistern on brain CT scan. 

## Discussion

Based on the results of present study, the survival rate of patients with very severe head trauma (GCS < 6) was 41.7%. The strong predictors of survival and 6-month favorable outcome of these patients were brisk pupillary reactivity and patent cistern on brain CT scans. 

The use of GCS is a universal method to classify the severity of patients with head injury; patients with GCS scores of 3-4-5 have limited survivability. These patients have poor prognosis. Prior studies reported an overall mortality of 42-87.5% (2-6). 

In literature, clinical characteristics and imaging findings have been shown to correlate with outcome in the very severe group. Nevertheless, limited numbers of studies have examined the outcomes of patients with GCS scores of 3-5. Bilateral fixed dilated pupils (BFDP) are a significant predictor in concordance with several studies (4, 13-16). 

Lieberman et al. studied 137 trauma patients with a GCS score of 3. Mortality was 100% in the BFDP group whereas the non-BFDP group had 77% mortality (14). Sakas et al. studied 40 trauma patients with BFDP. Mortality was 100% if patients presented any of the following features: surgery 6 or more hours after bilateral loss of pupil reactivity, age more than 65 years, or absent motor response (15). Tien et al. compared trauma patients with a GCS of 3 and BFDP with patients with a GCS score of 3 and reactive pupils. Mortality was 100% versus 42%; hemodynamic instability, midline shift, and herniation were observed in the BFDP patients (4). 

Generally, the neuronal pathway of pupillary light reflex is located in the intrinsic brainstem. Light reflex is conducted by optic tract fibers, which synapse in the pretectal area. The pretectal neurons synapse to the Edinger-Westphal complex. Finally, the ciliary nerve serves pupillary constrictor responses (16). Pupillary dilatation and no response to light are caused by a compressed ipsilateral oculomotor nerve and brainstem ischemia. Ritter et al. reported that brain stem blood flow (BBF) was 30.5+/-16.8 ml/100 g/min in BFDP patients whereas it was 43.8+/-18.7 ml/100 g/min (p< 0.001)(17). Therefore, the present study has quite a different classification in pupillary reactivity. Previous studies assessed light reflex of both pupils. We hypothesized that brisk reactivity of either one pupil or both pupils is sufficient to indicate the function of the brainstem. In addition, we found that only one pupil showing good response to light directly related to the chance of survival and favorable outcome.

**Table 1:** Baseline characteristics of studied patients

**Characteristic**	**N (%)**
**Sex**	
Male	83 (80.6)
Female	20 (19.4)
**Age (year)**	
16-20	11 (10.7)
21-40	50 (48.5)
41-60	31 (30.1)
>60	11 (10.7)
**Underlying disease**	
None	83 (80.6)
Hypertension	10 (9.9)
Neurological	1 (1.0)
Gastro enteric	1 (1.0)
Respiratory	1 (1.0)
Other	5 (4.9)
**Drug **	
None	92 (92.0)
Unidentified drug	3 (3.0)
Aspirin	1 (1.0)
Other	4 (4.0)
**Mechanism of trauma**	
Falling	7 (6.8)
Vehicle accident	8 (7.8)
Motorcycle accident	78 (75.7)
Pedestrians	3 (2.9)
Body assault	2 (1.9)
Other	5 (4.9)
**Glasgow coma scale**	
3	64 (62.1)
4	28 (27.2)
5	11 (10.7)
**Pupillary reactivity** *	
Poor pupils	79 (76.7)
Brisk pupil/pupils	22 (21.8)
**Motor response**	
M1	68 (66.0)
M2	24 (23.3)
M3	11 (10.7)
**Hypoxia episode**	18 (17.5)
**Hypotension episode**	46 (44.7)
**Calvarial skull fracture**	
None	42 (40.8)
Linear	57 (55.3)
Depressed	3 (2.9)
Compound	1 (1.0)
**Basal skull fracture**	60 (58.3)
**Intracranial findings**	
Cerebral contusion	56 (54.4)
Intracerebral hemorrhage	48 (46.6)
Subdural hemorrhage	78 (75.7)
Epidural hemorrhage	19 (18.4)
Intraventricular hemorrhage	20 (19.4)
Hydrocephalus	17 (16.5)
Brainstem hemorrhage	4 (3.9)
**Appearance of basal cistern **	
Patent	44 (43.1)
Obliteration	58 (56.3)
**Marshall classification**	
Grade I	5 (4.9)
Grade II	19 (18.4)
Grade III	32 (31.1)
Grade IV	1 (1)
Evacuated mass lesion	39 (37.9)
Non-evacuated mass lesion	7 (6.8)
**Operation **	
No	56 (54.4)
Craniotomy with clot removal	8 (7.8)
Decompressive craniotomy with clot removal	33 (32.0)
ICP monitoring	6 (5.8)
**GOS at discharge**	
Dead	60 (58.3)
Vegetative state	12 (11.7)
Severe disability	4 (3.9)
Moderate disability	16 (15.5)
Good recovery	11 (10.7)
**GOS at 6 months **	
Dead	60 (58.3)
Vegetative state	10 (9.7)
Severe disability	4 (3.9)
Moderate disability	9 (8.7)
Good recovery	20 (19.7)
**Outcome at 6 months**	
Unfavorable (GOS 1-3)	74 (71.8)
Favorable (GOS 4-5)	29 (28.2)

* 2 patients could not be categorized into groups because of periorbital swelling on both sides. GOS: Glasgow outcome scale.

**Table 2:** Associated factors of 6 months survival based on univariate analysis

**Factor**	**Death**	**Survival **	**OR (95%CI)**	***p ***
**Age (year)**				
< 55	47 (78.3)	37 (86.0)	Ref	0.32
> 55	13 (21.7)	6 (14.0)	0.58 (0.20-1.69)	
**Sex**				
Male	49 (81.7)	34 (79.1)	Ref	0.74
Female	11 (18.3)	9 (20.9)	1.17 (0.44-3.15)	
**Glasgow coma scale**				
3	40 (66.7)	24 (55.8)	Ref	0.26
4	16 (26.7)	12 (27.9)	1.25 (0.50-3.08)	
5	4 (6.7)	7 (16.3)	2.91 (0.77-11.01)	
**Pupillary reactivity**				
Poor pupils	53 (89.8)	26 (61.9)	Ref	0.001‡
Brisk pupil/pupils	6 (10.2)	16 (38.1)	6.16 (2.22-17.07)	
**Motor response **				
M1	42 (70.0)	26 (60.5)	Ref	0.28
M2	14 (23.3)	10 (23.3)	1.15 (0.44-2.97)	
M3	4 (6.7)	7 (16.3)	2.82 (0.75-10.60)	
**Hypoxia **				
No	47 (78.3)	38 (88.4)	Ref	0.18
Yes	13 (21.7)	5 (11.6)	0.49 (0.15-1.43)	
**Hypotension**				
No	27 (45.0)	30 (69.8)	Ref	0.01‡
Yes	33 (55.0)	13 (30.2)	0.33 (0.15-0.81)	
**Intracerebral hemorrhage (ICH)**				
No	32 (53.3)	22 (52.4)	Ref	0.92
Yes	28 (46.7)	20 (47.6)	1.03 (0.42-2.28)	
**Contusion**				
No	29 (48.3)	18 (41.9)	Ref	0.51
Yes	31 (51.7)	25 (58.1)	1.29 (0.59-2.86)	
**Posterior fossa ICH**				
No	59 (98.3)	40 (95.2)	Ref	0.36
Yes	1 (1.7)	2 (4.8)	1.26 (0.11-14.54)	
**Subdural hematoma thickness (cm)**				
< 1	41(68.3)	30 (71.4)	Ref	0.73
> 1	19 (31.7)	12 (28.6)	0.86 (0.36-2.04)	
**Epidural hematoma thickness (cm)**				
< 1.5	58 (96.7)	38 (90.5)	Ref	0.19
> 1.5	2 (3.3)	4 (9.5)	3.05 (0.53-17.49)	
**Intraventricular hemorrhage**				
No	45 (75.0)	37 (88.1)	Ref	0.10
Yes	15 (25.0)	5 (11.9)	0.40 (0.13-1.22)	
**Hydrocephalus**				
No	46 (76.7)	39 (92.9)	Ref	0.03‡
Yes	14 (23.3)	3 (7.1)	0.23 (0.68-0.94)	
**Midline shift (mm)**				
<5	39 (66.1)	27 (64.3)	Ref	0.85
> 5	20 (33.9)	15 (35.7)	1.08 (0.42-2.48)	
**Basal cistern appearance**				
Obliteration	40 (66.7)	18 (42.9)	Ref	0.01‡
Patent	20 (33.3)	24 (47.1)	2.66 (1.18-6.01)	
**Brainstem hemorrhage**				
No	58 (96.7)	40 (95.2)	Ref	1.45
Yes	2 (3.3)	2 (4.8)	1.45 (0.19-10.72)	
**Operation**				
No	39 (65.0)	17 (39.5)	Ref	0.01‡
Yes	21 (35.0)	26 (60.5)	2.84 (1.24-6.28)	

* *p* value of Chi-square test,

† *p* value of Fisher’s exact test,

‡ Statistical significant. OR: odds ratio, CI: confidence interval.

**Table 3:** Associated factors of 6 months survival based on multivariate analysis

**Variable**	**OR (95%CI)**	***p***
**Pupillary reactivity**		
Poor pupils	Ref	0.007
Brisk pupil/pupils	5.20 (1.57-17.26)	
**Basal cistern appearance **		
Obliteration	Ref	0.02
Patent	3.65 (1.22-10.91)	
**Hypotension**		
No	Ref	0.63
Yes	1.31 (0.42-4.07)	
**Hydrocephalus**		
No	Ref	0.82
Yes	1.18 (0.26-5.29)	
**Operation**		
No	Ref	0.36
Yes	0.58 (0.18-1.86)	

OR: odds ratio; CI: confident interval.

**Table 4:** Associated factors of 6 months favorable outcome based on Univariate analysis

**Factor**	**Unfavorable**	**Favorable**	**OR (95%CI)**	***p ***
**Age**				
< 55	58 (78.4)	26 (89.7)	Ref	0.26 †
> 55	16 (21.6)	3 (10.3)	0.41 (0.11-1.56)	
**Sex**				
Male	58 (78.4)	25 (86.2)	Ref	0.36
Female	16 (21.6)	4 (13.8)	0.58 (0.17-1.91)	
**GCS**				
3	48 (64.9)	16 (55.2)	Ref	0.63
4	19 (25.7)	9 (31.0)	1.42 (0.53-3.76)	
5	7 (9.5)	4 (13.8)	1.71 (0.44-6.62)	
**Pupillary reactivity**				
Poor pupils	64 (87.7)	15 (53.6)	Ref	0.001‡
Brisk pupil/pupils	9 (12.3)	13 (46.4)	6.2 (2.22-17.07)	
**Motor response**				
M1	51 (68.9)	17 (58.6)	Ref	0.60
M2	16 (21.6)	8 (27.6)	1.50 (0.54-4.12)	
M3	7 (9.5)	4 (13.8)	1.71 0.44-6.58)	
**Hypoxia **				
No	60 (81.1)	25 (86.2)	Ref	0.53
Yes	14 (18.9)	4 (13.8)	0.68 (0.25-2.28)	
**Hypotension**				
No	39 (52.7)	18 (62.1)	Ref	0.59
Yes	35 (47.3)	11 (37.9)	0.68 (0.28-1.63)	
**Intracranial hemorrhage (ICH)**				
No	41 (56.2)	13 (44.8)	Ref	0.30
Yes	32 (43.8)	16 (55.2)	1.57 (0.66-3.74)	
**Contusion**				
No	36 (48.6)	11 (37.9)	Ref	0.32
Yes	38 (51.4)	18 (62.1)	1.55 (0.64-3.72)	
**Posterior fossa ICH**				
No	71 (97.3)	28 (96.6)	Ref	0.84*
Yes	2 (2.7)	1 (3.4)	1.3 (0.11-14.54)	
**Subdural hemorrhage thickness (cm)**				
< 1	49 (66.2)	22 (78.6)	Ref	0.22
> 1	25 (33.8)	6 (21.4)	0.5 (0.19-1.48)	
**Epidural hemorrhage thickness (cm)**				
< 1.5	72 (97.3)	24 (85.7)	Ref	0.02‡
> 1.5	2 (2.7)	4 (14.3)	6.0 (1.03-34.84)	
**Intraventricular hemorrhage**				
No	57 (77.0)	25 (89.3)	Ref	0.16
Yes	17 (23.0)	3 (10.7)	0.40 (0.10-1.49)	
**Hydrocephalus**				
No	60 (81.1)	25 (89.3)	Ref	0.18
Yes	14 (18.9)	3 (10.7)	0.51 (0.13-1.94)	
**Midline shift (mm)**				
<5	46 (62.2)	22 (75.9)	Ref	0.18
> 5	28 (37.8)	7 (24.1)	0.52 (0.19-1.38)	
**Basal cistern appearance **				
Obliteration	49 (66.2)	9 (32.1)	Ref	0.02‡
Patent	25 (33.8)	19 (67.9)	4.1 (1.6-10.5)	
**Brainstem hemorrhage**				
No	71 (95.9)	27 (96.4)	Ref	0.91
Yes	3 (4.1)	1 (3.6)	0.87 (0.08-8.79)	
**Operation**				
No	40 (54.1)	16 (55.2)	Ref	0.98
Yes	34 (45.9)	13 (44.8)	0.95 (0.40-2.26)	

* *p* value of Chi-square test,

† *p* value of Fisher’s exact test,

‡ Statistical significant. OR: odds ratio, CI: confident interval.

**Table 5:** Associated factors of 6 months favorable outcome based on multivariate analysis

**Variable**	**OR (95%CI)**	***p*** ** value**
**Pupillary reactivity**		
Poor pupils	Ref	0.01
Brisk pupil/pupils	4.07(1.35-12.24)	
**Basal cistern appearance**		
Obliteration	Ref	0.02
Patent	3.54 (1.22-10.26)	
**Epidural hematoma thickness (cm)**		
<1.5	Ref	0.13
> 1.5	4.46 (0.64-31.15)	

CI: confident interval, OR: odds ratio.

* The number of patients was 101 patients because 2 patients with severe periborbital swelling on both sides could not have their pupils evaluated.

Abbrevation; NA: Not availiable.

In our study, another factor, the appearance of basal cistern on brain CT scan, has been determined to correlate with survival and outcome. Absence of obliterated basal cistern had an independent association with survival and good outcome. Toutant et al. studied 218 patients with severe head injury and found a strong relationship between mortality rate and death. In detail, death rate in patients with compressed cisterns was approximately twice as high compared with normal cistern (39% and 22%, respectively) (18). On the other hand, Mauritz et al. investigated outcomes of very severe head injury cases that had a GCS score of 3. The opening cistern on the first brain CT scan was one potential predictor of survival and good functional recovery (13).

The strength of the present study includes more detailed assessment of statistically significant factors affecting survival such as pupillary reactivity. However, the present study has limitations including retrospective study design and assessment of early outcome. Firstly, the quality of data and bias may be affected by study design. The second limitation is only assessing early outcome. Long-term outcome is an uncommon aspect to assess in these patients. Interestingly, late outcome assessment of survivors who had very severe head injuries in other studies has shown that physical and psychological impairment remains in many cases (19). For studying clinical implications, we suggest a prospective study design for better data quality. Long-term outcome assessment should also be considered in the future. It seems that very severe head trauma patients still have a reasonable chance to survive and management should be continued. The results of this study may guide physicians to decide whether to continue treatment in patients or not.

## Conclusion:

Based on the results of present study, the survival rate of patients with very severe head trauma (GCS < 6) was 41.7%. The strong predictors of survival and 6-month favorable outcome of these patients were brisk pupillary reactivity and patent cistern on brain CT scan. It seems that very severe head trauma patients still have a reasonable chance to survive and management should be continued.

## Authors’ contributions:

All authors passed four criteria for authorship contribution based on recommendations of the International Committee of Medical Journal Editors. 

## References

[1] Phuenpathom N, Tiensuwan M, Ratanalert S, Saeheng S, Sripairojkul B (2000). The changing pattern of head injury in Thailand. Journal of clinical neuroscience.

[2] Gennarelli TA, Spielman GM, Langfitt TW, Gildenberg PL, Harrington T, Jane JA (1982). Influence of the type of intracranial lesion on outcome from severe head injury: a multicenter study using a new classification system. Journal of neurosurgery.

[3] Jain S, Dharap SB, Gore MA (2008). Early prediction of outcome in very severe closed head injury. Injury.

[4] Tien HC, Cunha JR, Wu SN, Chughtai T, Tremblay LN, Brenneman FD (2006). Do trauma patients with a Glasgow Coma Scale score of 3 and bilateral fixed and dilated pupils have any chance of survival?. Journal of Trauma and Acute Care Surgery.

[5] Quigley MR, Vidovich D, Cantella D, Wilberger JE, Maroon JC, Diamond D (1997). Defining the limits of survivorship after very severe head injury. Journal of Trauma and Acute Care Surgery.

[6] Demetriades D, Kuncir E, Velmahos GC, Rhee P, Alo K, Chan LS (2004). Outcome and prognostic factors in head injuries with an admission Glasgow Coma Scale score of 3. Archives of Surgery.

[7] Chaudhuri K, Malham GM, Rosenfeld JV (2009). Survival of trauma patients with coma and bilateral fixed dilated pupils. Injury.

[8] Brazinova A, Mauritz W, Leitgeb J, Wilbacher I, Majdan M, Janciak I (2010). Outcomes of patients with severe traumatic brain injury who have Glasgow Coma Scale scores of 3 or 4 and are over 65 years old. Journal of neurotrauma.

[9] Marshall L, Marshall SB, Klauber M, Van Berkum CM, Eisenberg H, Jane J (1992). The diagnosis of head injury requires a classification based on computed axial tomography. Journal of neurotrauma.

[10] Jennett B, Bond M (1975). Assessment of outcome after severe brain damage: a practical scale. The Lancet.

[11] Teasdale G, Jennett B (1974). Assessment of coma and impaired consciousness: a practical scale. The Lancet.

[12] Free software of the R-Project group from the Department of Statistics and Mathematics of the Vienna University of Economics and Business Administration.

[13] Mauritz W, Leitgeb J, Wilbacher I, Majdan M, Janciak I, Brazinova A (2009). Outcome of brain trauma patients who have a Glasgow Coma Scale score of 3 and bilateral fixed and dilated pupils in the field. European Journal of Emergency Medicine.

[14] Lieberman JD, Pasquale MD, Garcia R, Cipolle MD, Li PM, Wasser TE (2003). Use of admission Glasgow Coma Score, pupil size, and pupil reactivity to determine outcome for trauma patients. Journal of Trauma and Acute Care Surgery.

[15] Sakas DE, Bullock MR, Teasdale GM (1995). One-year outcome following craniotomy for traumatic hematoma in patients with fixed dilated pupils. Journal of neurosurgery.

[16] Hultborn H, Mori K, Tsukahara N (1978). The neuronal pathway subserving the pupillary light reflex. Brain Research.

[17] Ritter AM, Muizelaar JP, Barnes T, Choi S, Fatouros P, Ward J (1999). Brain stem blood flow, pupillary response, and outcome in patients with severe head injuries. Neurosurgery.

[18] Toutant SM, Klauber MR, Marshall LF, Toole BM, Bowers SA, Seelig JM (1984). Absent or compressed basal cisterns on first CT scan: ominous predictors of outcome in severe head injury. Journal of neurosurgery.

[19] Thomsen IV (1984). Late outcome of very severe blunt head trauma: a 10-15 year second follow-up. Journal of Neurology, Neurosurgery & Psychiatry.

